# Association between cardiovascular, psychotropic and anti-inflammatory/analgesic drug use and vascular dysfunction in individuals with long COVID. BioICOPER study

**DOI:** 10.3389/fcvm.2025.1691153

**Published:** 2026-01-12

**Authors:** Silvia Arroyo-Romero, Leticia Gómez-Sánchez, Nuria Suárez-Moreno, Alicia Navarro-Cáceres, Andrea Domínguez-Martín, Cristina Lugones-Sánchez, Susana González-Sánchez, Andrea Sánchez-Moreno, Emiliano Rodríguez-Sánchez, Luis García-Ortiz, Elena Navarro-Matias, Manuel A. Gómez-Marcos

**Affiliations:** 1 Primary Care Research Unit of Salamanca (APISAL), Salamanca Primary Care Management, Institute of Biomedical Research of Salamanca (IBSAL), Salamanca, Spain; 2 Castilla and León Health Service-SACYL, Regional Health Management, Salamanca, Spain; 3 Emergency Service, University Hospital of La Paz P. of Castellana, Madrid, Spain; 4 Research Network on Chronicity, Primary Care and Health Promotion (RICAPPS), Salamanca, Spain; 5 Department of Medicine, University of Salamanca, Salamanca, Spain; 6 Department of Biomedical and Diagnostic Sciences, University of Salamanca, Salamanca, Spain

**Keywords:** drug use, long COVID, arterial stiffness, vascular structure, cardiovascular risk

## Abstract

**Introduction:**

While the deterioration in the general health of patients with long COVID (LC) is well documented, no studies have assessed changes in medication use and their relationships with vascular health. This study aimed to evaluate the increase in the use of various drug classes in LC and its relationship with vascular structure and function.

**Methods:**

Each participant in the sample of 305 subjects diagnosed with LC completed a questionnaire on medication use, verified in medical records. Pre-pandemic and current drug use were recorded. Arterial stiffness was measured with the VaSera device, which estimates the cardio-ankle vascular index and brachial-ankle pulse wave velocity (ba-PWV); carotid-femoral pulse wave velocity was determined using the Sphygmocor device. Vascular structure was assessed by carotid intima-media thickness (c-IMT), measured with a Sonosite Micromax ultrasound. This analysis focuses exclusively on macrovascular parameters. Statistical analyses were performed with SPSS software.

**Results:**

Use of all classes of medication increased. Patients with a greater rise in drug use after an LC diagnosis showed higher vascular parameters. Greater cardiovascular drug use was positively associated with ba-PWV, an indicator of arterial stiffness (*β* = 0.301, 95%CI: 0.024–0.577). Increased anti-inflammatory/analgesic drug use was positively associated with c-IMT, a marker of vascular wall thickness (*β* = 0.012, 95%CI: 0.001–0.023).

**Conclusions:**

Medication use rose from 2019 to the time of inclusion in the study. The increase in cardiovascular and anti-inflammatory/analgesic drug use was positively associated with ba-PWV and c-IMT, respectively, suggesting a link between greater drug use and impaired vascular health in LC.

## Highlights

• Long COVID is associated with impaired vascular structure and function in a well-defined post-COVID cohort.
• Vascular alterations persist despite pharmacological treatment, highlighting unmet therapeutic needs.
• Increased drug use reflects the physical and mental health burden in individuals with Long COVID.
• This is the first study to investigate the relationship between drug consumption and vascular parameters in Long COVID.

## 1 Introduction

The health landscape has been transformed by the COVID-19 pandemic, not just because of the severity of the acute infection, but also because of the long-term symptoms, a condition known as long COVID (LC) (1). The pathophysiology of LC is multifactorial and unknown, but chronic inflammation and endotheliopathy stand out (2). These mechanisms boost the progression of pre-existing chronic diseases and the emergence of new pathologies, affecting both cardiovascular (3) and mental health (4). Specifically, LC has been linked to an increase in cardiovascular risk factors such as hypertension (5),dyslipidemia (6),diabetes mellitus (DM) (7) or increased risk of thrombotic events (8). In addition, a higher incidence of psychiatric pathologies such as depression or anxiety has been reported in patients with LC (9). The growth in these pathologies and the need to control the persistent symptoms of the disease [more than 200 symptoms have been described (10)] gives rise to greater use of medications and health resources among LC patients. This increased drug use is more pronounced than in healthy individuals (11). Different therapy options have been proposed for the different effects of LC (12). To combat chronic inflammation, patients with LC use more anti-inflammatory and immunomodulatory drugs (13). The presence of anxious-depressive symptoms, dysautonomia or chronic fatigue generate increased use of antihypertensive drugs (14), antihyperlipidaemics (15), antidepressants and anxiolytics (16). Polypharmacy in patients with LC reflects symptom variability, the multifactorial nature of the disease, and the limited knowledge about effective treatments, all of which increases the risk of overtreatment and iatrogenesis (17).

Vascular alteration (18) and cardiovascular risk factors (19) are key determinants in the development of LC. Controlling cardiovascular risk factors in the acute phase of COVID-19 has been linked to a lower probability of LC developing or less severe symptoms if it does occur (19, 20). Vascular health analysis (vascular structure and function) is a predictor of cardiovascular risk (21). Vascular health can be assessed noninvasively by the following measures: carotid intima-media thickness (c-IMT), which assesses vascular structure (22); carotid-femoral pulse wave velocity (cf-PWV) for central arterial stiffness; brachial-ankle pulse wave velocity (ba-PWV) for peripheral arterial stiffness; and the cardio-ankle vascular index (CAVI), which estimates both central and peripheral arterial stiffness (23). The present study focuses exclusively on macrovascular parameters, assessed through validated non-invasive techniques.

Vascular damage (especially that produced in LC) is triggered by alterations in the renin-angiotensin-aldosterone system and inflammatory mediators (24). These signalling pathways are therapeutic targets for numerous medication groups. COVID-19 interacts with angiotensin-converting enzyme 2 (ACE2), leading to increased angiotensin II levels and promoting endothelial dysfunction, inflammation (25), and elevated blood pressure (26). The relationship between dyslipidemia and SARS-CoV-2 infection is bidirectional: elevated cholesterol may enhance viral entry through increased ACE2 expression (27), while the infection itself raises cholesterol and triglyceride levels and promotes atheromatous plaque formation through oxidative stress (15). Lipid-lowering therapy can counteract these effects by reducing vascular inflammation through the endothelial nitric oxide synthase/ nitric oxide (eNOS/NO) and Yes-associated protein/transcriptional coactivator with PDZ-binding motif (YAP/TAZ) pathways, stabilizing atherosclerotic plaque, and activating longevity genes such as Klotho (28). In DM, SARS-CoV-2 can cause direct β-cell injury and reduced insulin production (29), while insulin resistance and chronic inflammation favour the persistence of LC (30). Beyond glucose control, agents such as metformin modulate immune and oxidative stress responses and may even inhibit viral replication (31, 32). Persistent endothelial injury and microclot formation, together with complement dysregulation and an increased von Willebrand factor/ a disintegrin and metalloproteinase with thrombospondin type 1 motif member 13 (ADAMTS13) ratio, underlie the prothrombotic state characteristic of LC, which could be mitigated by antiplatelet or anticoagulant therapy (33, 34).

**Figure 1:**
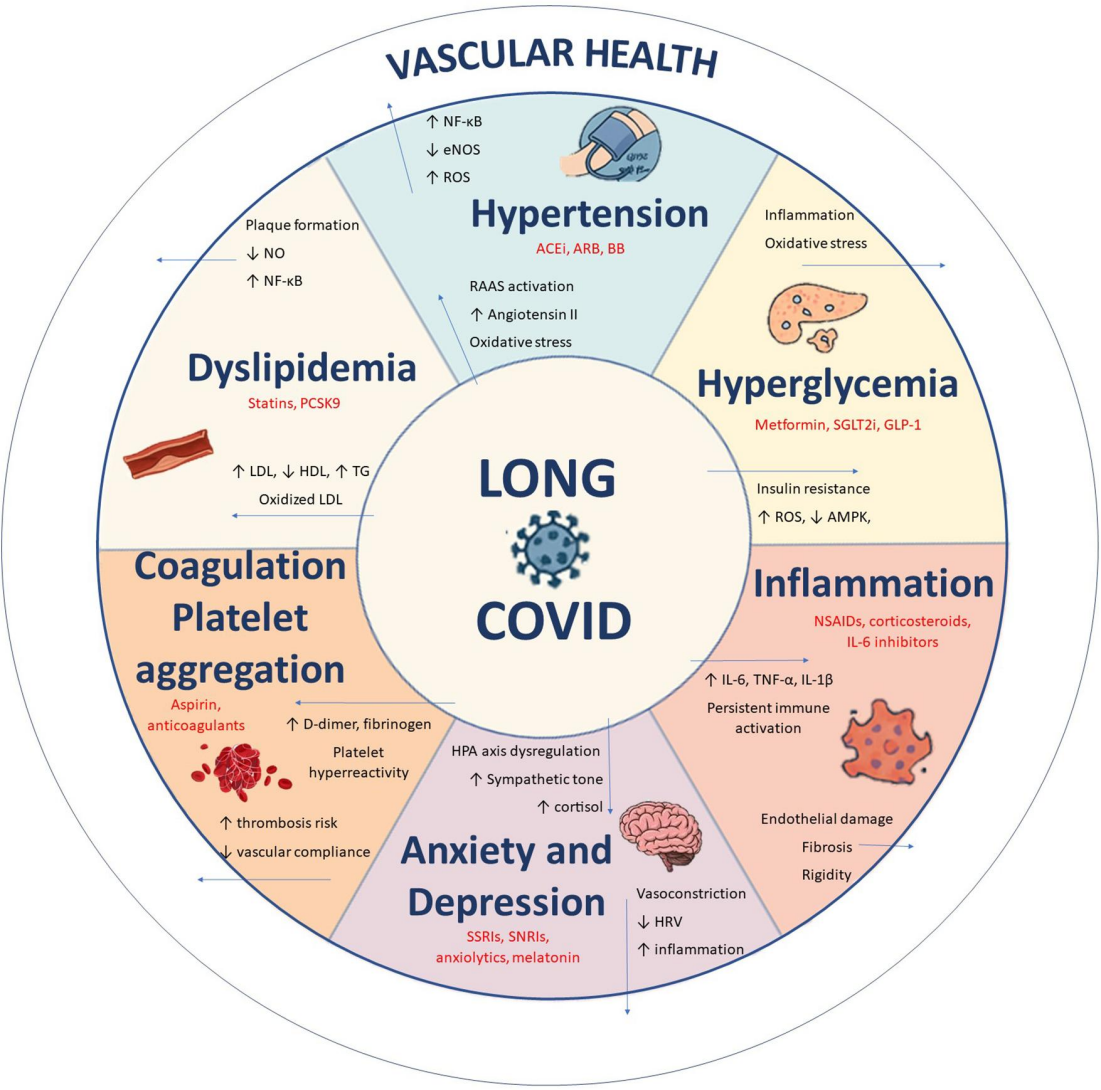
Pathophysiological pathways linking LC to multisystem alterations and vascular dysfunction. Source: own elaboration.

While the action of certain antihypertensives (35, 36), antihyperlipidaemics and hypoglycaemics on vascular health is well known (28)), other drugs, such as antidepressants and anxiolytics, also have off-target vascular effects (37). Treatment with fluvoxamine, in addition to its effects on anxiety and depressive symptoms, has shown favourable outcomes for LC, reducing viral load and the concentration of pro-inflammatory cytokines (38). The use of selective serotonin reuptake inhibitors (SSRIs) during acute infection has also been associated with a lower risk of developing LC (39). The “vascular depression” hypothesis proposes that microvascular disease, endothelial dysfunction, and vascular inflammation contribute to depressive symptoms, mechanisms that may overlap with the neuropsychiatric manifestations of LC (40, 41). Systemic inflammation and immune dysregulation are likewise central to LC pathophysiology, and early exposure to anti-inflammatory drugs during acute infection has been linked to a greater risk of persistent symptoms, highlighting the complex and bidirectional relationship between inflammation, drug use, and vascular health (42). Figure 1 shows the interrelationships between drug use, vascular health, and LC.

COVID-19 infection (43) and lifestyle choices (44) have been shown to influence vascular health in patients with LC, yet the relationship between vascular health and drug use in LC has not been evaluated. Further research is needed to clarify how medication use influences vascular outcomes in these patients.

## 2 Materials and methods

### 2.1 Objectives

The aims of this study were to analyse the increase in the use of cardiovascular, psychotropic and anti-inflammatory/analgesic drugs, its relationship with parameters of vascular structure and function in subjects diagnosed with LC, and the differences by sex.

### 2.2 Study design

This work is part of the BioICOPER study, a descriptive cross-sectional study conducted at the Salamanca Primary Care Research Unit. The study protocol has been previously published (45). The study was registered on ClinicalTrials.gov in April 2023 (registration number NCT05819840).

### 2.3 Study population

Using consecutive sampling, 305 participants diagnosed with LC were recruited throughout 2023 from the primary care registries and the LC clinic of Salamanca University Hospital's internal medicine department.

The inclusion criteria were:

1. A confirmed diagnosis of LC according to the World Health Organization (WHO) (46): symptoms that starts within three months of the acute phase of COVID-19, last for at least two months, cannot be explained by another diagnosis, and may persist, recur after initial recovery, or fluctuate over time (46).
2. Age ≥ 18 years.
3. Ability to complete the clinical assessments and questionnaires.

Exclusion criteria were:

1. Subjects excluded were those in a terminal state.
2. Established cardiovascular disease (ischemic heart disease or cerebrovascular event).
3. Severe chronic kidney disease (glomerular filtration rate below 30 mL/min/1.73 m^2^).
4. Inability to travel to the health centre for the evaluations.

### 2.4 Variables and measurement instruments

Four trained healthcare professionals administered the necessary examinations and questionnaires following a standardised protocol. The questionnaires included and assessment of previous and current drug consumption. An independent researcher was responsible for data quality control.

#### 2.4.1 Sociodemographic variables

Participants' age and sex were recorded at the moment of inclusion in the study.

#### 2.4.2 Drug use

The use of medication was assessed with a standardised questionnaire administered to each participant and subsequently verified in medical records, both prior to the illness (2019) and at the time of inclusion in the study (current). The increase in drug use was determined by evaluating those participants currently taking the medication group in question but who did not use it in 2019; that is, participants who started taking the drug after developing LC. The use of the following medications was recorded: cardiovascular drugs (antihypertensives, antihyperlipidaemics, hypoglycaemics, anticoagulants and platelet aggregation inhibitors), antidepressants and anxiolytics (benzodiazepines and hypnotics), and anti-inflammatory and analgesic drugs (non-steroidal anti-inflammatory drugs, oral corticosteroids, paracetamol, and other analgesics).

#### 2.4.3 Vascular structure and arterial stiffness

##### 2.4.3.1 Vascular structure

c-IMT was measured to assess vascular structure (47). c-IMT was determined with a Sonosite Micromaxx ultrasound system (FUJIFILM Sonosite, USA) with a high-resolution linear probe and Sonocal software. Automatic measurements were performed in accordance with a previously published protocol (48). A 10 mm section of the common carotid artery was selected, 1 cm from the bifurcation. Measurements of the proximal and distal walls of the carotid artery were taken from this section in anterior, lateral and posterior projections. Ultrasound images were obtained with the subject in the supine position, with the head hyperextended and tilted toward the contralateral shoulder. The staff responsible for the imaging were previously trained.

##### 2.4.3.2 Arterial stiffness

Arterial stiffness was assessed using cf-PWV, ba-PWV and CAVI. Cf-PWV was measured using the SphygmoCor device (AtCor Medical Pty Ltd, Head Office, Australia), with the participant in the supine position. The distance between the sternal jugulum and the sensor location on the carotid and radial arteries or carotid and femoral arteries was determined (49), and the time measurement was based on the delay of the carotid, radial and femoral artery pulse waves relative to the R wave of the electrocardiogram.

Ba-PWV and CAVI were analysed using the VaSera VS-2000 device (Fukuda Denshi Co., Ltd., Japan), with the patient silent and motionless. Electrodes were attached to the arms and ankles, and a heart sound microphone was placed in the second intercostal space. Ba-PWV was estimated using the following equation: ba-PWV = (0.5934 × height in cm + 14.4724)/tba, where tba refers to the interval between the detection of brachial waves and ankle waves (50). CAVI was estimated with the equation: stiffness parameter *β* = 2*ρ* × 1/(SBP-DBP) × ln(SBP/DBP) × PWV, where *ρ* is the blood density, SBP is the systolic blood pressure, DBP is the diastolic blood pressure and PWV is the pulse wave velocity determined between the aortic valve and the ankle. Measurements obtained after three consecutive heartbeats were considered valid (51).

#### 2.4.4 Cardiovascular risk factors

##### 2.4.4.1 Blood pressure measurement

SBP and DBP were measured in accordance with the European Society of Hypertension recommendations (52). After remaining seated for at least 5 min, three measurements were taken on the participant's dominant arm using an OMRON M10-IT sphygmomanometer (Omron Healthcare, Japan), with the average of the last two measurements being recorded. “Hypertension” was considered if the participants had blood pressure ≥ 140/90 mmHg or if they consumed antihypertensive drugs. 

##### 2.4.4.2 Analytical parameters

We determined the following analytical parameters: total cholesterol, low-density lipoprotein (LDL) cholesterol, high-density lipoprotein (HDL) cholesterol, triglycerides, and fasting plasma glucose (FPG). On the second visit, venous blood was drawn between 8:00 and 9:00 am, with subjects fasting, without having drunk alcohol or caffeine, or smoked in the previous 12 h. “Dyslipidaemia” was diagnosed if participants had total cholesterol levels ≥240 mg/dL, LDL ≥160 mg/dL, HDL <40 mg/dL in men and <50 mg/dL in women, or 153 triglycerides ≥150 mg/dL; or if they were taking lipid-lowering drugs. Subjects with FPC ≥126 mg/dL or those taking hypoglycaemic medication were classified as having “DM”.

##### 2.4.4.3. Anthropometric measurements

Participants were weighed (in kg) using the InBody 230 monitor (InBody Co., Ltd., South Korea), after fasting for 2 h, without shoes and wearing light clothing. Their height (cm) was measured standing barefoot on a stadiometer (Seca 222, Medical Scale and Measurement Systems, United Kingdom) and body mass index (BMI) was calculated by dividing weight in kg by height in metres squared (m2). “Obesity” was diagnosed if BMI was ≥ 30 kg/m^2^.

##### 2.4.4.4. Smoking status

To assess smoking status, we used a four-item questionnaire adapted from the WHO MONICA project (53). Participants were classified as active smokers or non-smokers.

### 2.5 Statistical analysis

The means of two-category quantitative variables were compared using Student's t-test, while the chi-square test was used to compare two categories of categorical variables. Spearman's rho correlation coefficient was used to correlate the relationship between measures of vascular structure and function and the increase in the use of different medications. Multiple regression models were applied to identify the association between increased drug use and vascular parameters. The vascular parameters used as dependent variables were c-IMT measured in mm, cf-PWV and ba-PWV measured in m/s, and CAVI. The increases in the use of cardiovascular medications, antidepressants/anxiolytics and anti-inflammatory/analgesic medications were taken as independent variables. Age in years and sex were considered as control variables. A *p* value <0.05 was regarded as statistically significant. Data were analysed using SPSS for Windows, v28.0 (IBM Corp., Armonk, USA).

### 2.6 Ethical principles

The study was approved by the Ethics Committee for Drug Research of the Salamanca Health Area on June 27, 2022 (CEIm reference code: Ref. PI 2022 06 1048), and the guidelines of the Declaration of Helsinki (54) and the WHO were followed throughout. Participant confidentiality was guaranteed at all times in accordance with Organic Law 3/2018, European Regulation 2016/679, and Council Regulation (EC) No. 27/04/2016 on Data Protection. All participants signed informed consent before being included in the study and after receiving information about the procedures involved.

## 3 Results

### 3.1 Participant characteristics

The mean time from infection to inclusion in the study was 38.7 ± 10.0 months overall, with no differences between men (38.5 ± 10.0 months) and women (38.7 ± 9.4 months), *p* = 0.10.

Table 1 shows the use of medication by participants prior to the pandemic (2019), overall and by sex. Before COVID-19 infection, men used significantly more cardiovascular drugs than women including antihypertensives (23.7% vs. 12.0%, *p* = 0.009) and antihyperlipidaemics (24.7% vs. 7.2%, *p* < 0.001). Conversely, women took more antidepressants and anxiolytics than men (antidepressants: 13% vs. 5.2%, *p* = 0.038; anxiolytics: 11.1% vs. 4.1%, *p* = 0.047). No differences were found in the use of hypoglycaemic agents, platelet aggregation inhibitors, anticoagulants, anti-inflammatory drugs or analgesics (all *p* > 0.05).

**Table 1:** Drug use in subjects with long COVID before the pandemic, overall and by sex.

Drugs	Overall (n° = 305)	Women (n° = 208)	Men (n° = 97)	*P*
Cardiovascular drugs, mean ± SD	0.42 ± 0.84	0.29 ± 0.69	0.70 ± 1.05	<0.001
Cardiovascular drugs, *n* (%)	79 (25.9%)	40 (19.2%)	39 (40.2%)	<0.001
Antihypertensives, mean ± SD	0.20 ± 0.52	0.15 ± 0.44	0.32 ± 0.65	0.011
Antihypertensives, *n* (%)	48 (15.7%)	25 (12.0%)	23 (23.7%)	0.009
Antihyperlipidaemics, mean ± SD	0.13 ± 0.35	0.08 ± 0.29	0.24 ± 0.45	0.001
Antihyperlipidaemics, *n* (%)	39 (12.8%)	15 (7.2%)	24 (24.7%)	<0.001
Hypoglycaemics, mean ± SD	0.07 ± 0.25	0.05 ± 0.21	0.10 ± 0.31	0.056
Hypoglycaemics, *n* (%)	20 (6.6%)	10 (4.8%)	10 (10.3%)	0.071
Platelet aggregation inhibitors/Anticoagulants, mean ± SD	0.03 ± 0.16	0.02 ± 0.14	0.04 ± 0.20	0.164
Platelet aggregation inhibitors/Anticoagulants, *n* (%)	8 (2.6%)	4 (1.9%)	4 (4.1%)	0.263
Antidepressants/Anxiolytics, mean ± SD	0.20 ± 0.52	0.25 ± 0.58	0.10 ± 0.37	0.004
Antidepressants/Anxiolytics, *n* (%)	46 (15.1%)	38 (18.3%)	8 (8.2%)	0.023
Antidepressants, mean ± SD	0.11 ± 0.31	0.13 ± 0.34	0.05 ± 0.22	0.008
Antidepressants *n* (%)	32 (10.5%)	27 (13.0%)	5 (5.2%)	0.038
Anxiolytics mean ± SD	0.10 ± 0.33	0.12 ± 0.35	0.05 ± 0.27	0.030
Anxiolytics *n* (%)	27 (8.9%)	23 (11.1%)	4 (4.1%)	0.047
Anti-inflammatory agents/Analgesics, mean ± SD	0.18 ± 0.50	0.20 ± 0.51	0.12 ± 0.46	0.092
Anti-inflammatory agents/Analgesics, *n* (%)	41 (13.4%)	33 (15.9%)	8 (8.2%)	0.069
Anti-inflammatory agents, mean ± SD	0.11 ± 0.31	0.12 ± 0.33	0.07 ± 0.26	0.084
Anti-inflammatory agents, *n* (%)	32 (10.5%)	25 (12.0%)	7 (7.2%)	0.202
Analgesics, mean ± SD	0.07 ± 0.34	0.08 ± 0.35	0.05 ± 0.30	0.233
Analgesics, *n* (%)	15 (4.9%)	12 (5.8%)	3 (3.1%)	0.314

Values are means and standard deviations for continuous data, and number and proportions for categorical data. SD, standard deviations.

*p* value: differences between men and women.

Table 2 shows the participants' general characteristics, comorbidities and their use of medication at the time of inclusion in the study. The mean age of the sample was 52.7 ± 11.9 years. Men showed a higher burden of cardiovascular risk factors, including SBP, DBP, triglycerides, FPG, body weight, BMI (all *p* < 0.001). Hypertension was present in 35.2% of the sample, dyslipidaemia in 66.3%, diabetes mellitus in 12.2%, obesity in 32.5%, and 5.7% of participants were active smokers. These comorbidities are specified to provide context for their potential influence on vascular measurements. Women showed higher levels of total cholesterol (*p* = 0.029) and HDL cholesterol (*p* < 0.001).

**Table 2:** Characteristics of the participants at the time of inclusion in the study, overall and by sex.

Variable	Overall (n° = 305)	Women (n° = 208)	Men (n° = 97)	*P*
Age, (years)	52.71 ± 11.94	51.32 ± 11.54	55.70 ± 12.28	0.001
Cardiovascular Risk Factors				
SBP, (mmHg)	119.95 ± 16.75	115.52 ± 15.94	129.45 ± 14.37	<0.001
DBP, (mmHg)	76.85 ± 11.11	74.30 ± 10.20	82.34 ± 11.04	<0.001
Hypertension, *n* (%)	109 (35.2)	57 (27.5)	52 (53.6)	<0.001
Total cholesterol, (mg/dL)	187.45 ± 34.30	189.95 ± 34.71	182.11 ± 32.94	0.029
LDL cholesterol, (mg/dL)	113.03 ± 31.76	112.77 ± 31.67	113.59 ± 32.12	0.417
HDL cholesterol, (mg/dL)	56.92 ± 13.58	60.73 ± 13.06	48.78 ± 10.86	<0.001
Triglycerides, (mg/dL)	102.23 ± 50.81	95.09 ± 47.52	117.47 ± 54.39	<0.001
Dyslipidaemia, *n* (%)	201 (66.3)	130 (63.1)	71 (73.2)	0.053
FPG, (mg/dL)	87.88 ± 17.67	84.84 ± 15.74	94.37 ± 19.78	<0.001
Diabetes Mellitus, *n* (%)	37 (12.2)	15 (7.3)	22 (22.7)	<0.001
Weight, kg	75.95 ± 17.39	70.29 ± 15.46	88.09 ± 14.95	<0.001
Height, cm	164.50 ± 8.71	160.77 ± 6.52	172.51 ± 7.35	<0.001
BMI, (kg/m^2^)	27.97 ± 5.55	27.21 ± 5.78	29.60 ± 4.64	<0.001
Obesity, *n* (%)	99 (32.5)	55 (26.4)	44 (45.4)	<0.001
Active smoker, *n* (%)	17 (5.7)	9 (4.5)	8 (8.4)	0.065
Vascular structure and function				
c-IMT, (mm)	0.64 ± 0.09	0.62 ± 0.07	0.68 ± 0.12	<0.001
cf-PWV, (m/s)	7.79 ± 2.55	7.30 ± 2.06	8.85 ± 3.13	<0.001
ba-PWV, (m/s)	12.79 ± 2.38	12.39 ± 2.26	13.63 ± 2.40	<0.001
CAVI	7.50 ± 1.27	7.32 ± 1.18	7.90 ± 1.36	<0.001
Drugs				
Cardiovascular drugs, mean ± SD	0.80 ± 1.19	0.61 ± 1.04	1.23 ± 1.38	<0.001
Cardiovascular drugs, *n* (%)	132 (43.3%)	70 (33.7%)	62 (63.9%)	<0.001
Antihypertensives, mean ± SD	0.36 ± 0.70	0.30 ± 0.66	0.49 ± 0.78	0.024
Antihypertensives, *n* (%)	79 (25.9%)	45 (21.6%)	34 (35.1%)	0.013
Antihyperlipidaemics, mean ± SD	0.29 ± 0.56	0.20 ± 0.48	0.47 ± 0.66	<0.001
Antihyperlipidaemics, *n* (%)	75 (24.6%)	35 (16.8%)	40 (41.2%)	<0.001
Hypoglycaemics, mean ± SD	0.11 ± 0.31	0.07 ± 0.25	0.19 ± 0.39	0.004
Hypoglycaemics, *n* (%)	32 (10.5%)	14 (6.7%)	18 (18.6%)	0.002
Platelet aggregation inh/Anticoagulants, mean ± SD	0.05 ± 0.22	0.04 ± 0.19	0.08 ± 0.28	0.080
Platelet aggregation inh/Anticoagulants, *n* (%)	16 (5.2%)	8 (3.8%)	8 (8.2%)	0.108
Antidepressants/Anxiolytics mean ± SD	0.49 ± 0.74	0.57 ± 0.79	0.30 ± 0.58	<0.001
Antidepressants/Anxiolytics *n* (%)	106 (34.8%)	83 (39.9%)	23 (23.7%)	0.006
Antidepressants, mean ± SD	0.25 ± 0.43	0.30 ± 0.46	0.14 ± 0.35	0.001
Antidepressants *n* (%)	76 (24.9%)	62 (29.8%)	14 (14.4%)	0.004
Anxiolytics mean ± SD	0.24 ± 0.46	0.27 ± 0.49	0.16 ± 0.39	0.012
Anxiolytics *n* (%)	67 (22.0%)	53 (25.5%)	14 (14.4%)	0.030
Anti-inflammatory agents/Analgesics, mean ± SD	0.49 ± 0.77	0.55 ± 0.80	0.34 ± 0.68	0.017
Anti-inflammatory agents/Analgesics, *n* (%)	104 (34.1%)	80 (38.5%)	24 (24.7%)	0.019
Anti-inflammatory agents, mean ± SD	0.26 ± 0.47	0.31 ± 0.50	0.16 ± 0.39	0.001
Anti-inflammatory agents, *n* (%)	76 (24.9%)	62 (29.8%)	14 (14.4%)	0.004
Analgesics, mean ± SD	0.22 ± 0.50	0.24 ± 0.52	0.19 ± 0.46	0.188
Analgesics, *n* (%)	56 (18.4%)	41 (19.7%)	15 (15.5%)	0.372

Values are means and standard deviations for continuous data, and number and proportions for categorical data. SBP, systolic blood pressure; DBP, diastolic blood pressure; FPG, fasting plasma glucose; LDL-C, low-density lipoprotein cholesterol; HDL-C, high–density lipoprotein cholesterol; BMI, body mass index; c-IMT, intima–media thickness of common carotid; cf-PWV, carotid-femoral pulse wave velocity; ba-PWV, brachial-ankle pulse wave velocity; CAVI, cardio-ankle vascular index.

*p* value: differences between men and women.

Men presented significantly worse vascular parameters: higher cf-PWV (men: 8.85 ± 3.13 vs. women: 7.30 ± 2.06), ba-PWV (men: 13.63 ± 2.40 vs. women: 12.39 ± 2.26), and CAVI values (men: 7.90 ± 1.36 vs. women: 7.32 ± 1.18) (all *p* < 0.001), as well as slightly higher c-IMT (women 0.62 ± 0.07 mm vs. men 0.68 ± 0.12 mm, *p* < 0.001) (Table 2).

At the time of inclusion, men continued to use more cardiovascular drugs than women (antihypertensives *p* = 0.024; antihyperlipidaemics *p* < 0.001; hypoglycaemics *p* = 0.004). Women had a greater use of antidepressants (*p* = 0.001), anxiolytics (*p* = 0.012), and anti-inflammatory agents (*p* = 0.001). No sex differences were found in antiplatelet/anticoagulant drug use (*p* > 0.05) or analgesic use (*p* > 0.05) (Table 2).

### 3.2 Increase in drug use

 Table 3 shows the increase in the use of medications from the pre-pandemic period to the time subjects were included in the study, both overall and by sex. All drug groups saw increased use. While men had a greater increase in the use of cardiovascular drugs: antihyperlipidaemics and hypoglycaemics (an increase of 21.6% and 8.2%, respectively, *p* < 0.05)), the increase in the use of anxiolytics was more pronounced in women (an increase of 24.7% *p* = 0.038). These findings highlight a global rise in medication consumption after LC onset, with sex-specific patterns depending on therapeutic class.

**Table 3:** Increase in drug use between the two measurements, overall and by sex (current-2019).

Drugs	Global	Women	Men	*P*
Cardiovascular drugs, mean ± SD	0.38 ± 0.77	0.31 ± 0.67	0.53 ± 0.94	0.023
Cardiovascular drugs, *n* (%)	88 (28.9%)	50 (24.0%)	38 (39.2%)	0.007
Antihypertensives, mean ± SD	0.16 ± 0.48	0.15 ± 0.45	0.17 ± 0.55	0.426
Antihypertensives, *n* (%)	48 (15.7%)	30 (14.4%)	18 (18.6%)	0.356
Antihyperlipidaemics, mean ± SD	0.16 ± 0.46	0.12 ± 0.41	0.24 ± 0.56	0.033
Antihyperlipidaemics, *n* (%)	44 (14.4%)	23 (11.1%)	21 (21.6%)	0.014
Hypoglycaemics, mean ± SD	0.04 ± 0.20	0.02 ± 0.14	0.08 ± 0.28	0.018
Hypoglycaemics, *n* (%)	12 (3.9%)	4 (1.9%)	8 (8.2%)	0.008
Platelet aggregation inhibitors/Anticoagulants, mean ± SD	0.03 ± 0.21	0.02 ± 0.20	0.04 ± 0.25	0.201
Platelet aggregation inhibitors/Anticoagulants, *n* (%)	11 (3.6%)	6 (2.9%)	5 (5.2%)	0.322
Antidepressants/Anxiolytics mean ± SD	0.28 ± 0.60	0.32 ± 0.65	0.20 ± 0.47	0.028
Antidepressants/Anxiolytics *n* (%)	73 (24.3%)	57 (27.9%)	16 (16.5%)	0.030
Antidepressants, mean ± SD	0.14 ± 0.40	0.17 ± 0.42	0.09 ± 0.33	0.044
Antidepressants *n* (%)	49 (16.1%)	39 (18.8%)	10 (10.3%)	0.062
Anxiolytics mean ± SD	0.14 ± 0.36	0.15 ± 0.38	0.10 ± 0.31	0.106
Anxiolytics *n* (%)	73 (23.9%)	57 (27.4%)	16 (16.5%)	0.038
Anti-inflammatory agents/Analgesics, mean ± SD	0.31 ± 0.72	0.35 ± 0.74	0.22 ± 0.68	0.060
Anti-inflammatory agents/Analgesics, *n* (%)	80 (26.2%)	60 (28.8%)	20 (20.6%)	0.128
Anti-inflammatory agents, mean ± SD	0.16 ± 0.45	0.19 ± 0.47	0.08 ± 0.40	0.018
Anti-inflammatory agents, *n* (%)	80 (26.2%)	60 (28.8%)	20 (20.6%)	0.128
Analgesics, mean ± SD	0.15 ± 0.46	0.16 ± 0.47	0.13 ± 0.45	0.333
Analgesics, *n* (%)	45 (14.8%)	32 (15.4%)	13 (13.4%)	0.649

Values are means and standard deviations for continuous data, and number and proportions for categorical data.

*p* value: differences between men and women.

### 3.3 Vascular structure and function in relation to increased drug use

As shown in Table 4, participants with increased use of cardiovascular drugs presented significantly higher vascular structure and function parameters. In this group, c-IMT (0.66 ± 0.07 vs. 0.63 ± 0.10 mm; *p* = 0.003), cf-PWV (8.68 ± 3.02 vs. 7.43 ± 2.24 m/s; *p* < 0.001), ba-PWV (13.76 ± 2.37 vs. 12.40 ± 2.27 m/s; *p* < 0.001) and CAVI (7.89 ± 1.19 vs. 7.34 ± 1.26; *p* < 0.001) were all significantly elevated.

**Table 4:** Vascular parameter values in subjects with and without increased drug use.

c-IMT (mm)	Increase	No increase	*P*
Cardiovascular Drugs, mean ± SD	0.66 ± 0.07	0.63 ± 0.10	0.003
Antihypertensives, mean ± SD	0.66 ± 0.08	0.63 ± 0.10	0.020
Antihyperlipidaemics, mean ± SD	0.67 ± 0.07	0.63 ± 0.09	0.006
Hypoglycaemics, mean ± SD	0.67 ± 0.07	0.64 ± 0.09	0.108
Platelet aggregation inhibitors/Anticoagulants, mean ± SD	0.64 ± 0.07	0.64 ± 0.09	0.470
Antidepressants/Anxiolytics mean ± SD	0.63 ± 0.07	0.64 ± 0.10	0.195
Antidepressants, mean ± SD	0.63 ± 0.08	0.64 ± 0.10	0.159
Anxiolytics mean ± SD	0.63 ± 0.07	0.64 ± 0.10	0.223
Anti-inflammatory agents/Analgesics, mean ± SD	0.65 ± 0.12	0.63 ± 0.08	0.068
Anti-inflammatory agents, mean ± SD	0.65 ± 0.12	0.63 ± 0.08	0.068
Analgesics, mean ± SD	0.66 ± 0.11	0.64 ± 0.09	0.063
cf-PWV (m/s)			
Cardiovascular drugs, mean ± SD	8.68 ± 3.02	7.43 ± 2.24	<0.001
Antihypertensives, mean ± SD	8.73 ± 3.18	7.62 ± 2.38	0.013
Antihyperlipidaemics, mean ± SD	8.59 ± 2.58	7.65 ± 2.52	0.012
Hypoglycaemics, mean ± SD	9.88 ± 3.02	7.70 ± 2.50	0.002
Platelet aggregation inhibitors/Anticoagulants, mean ± SD	7.01 ± 2.13	7.82 ± 2.56	0.151
Antidepressants/Anxiolytics mean ± SD	7.91 ± 2.74	7.69 ± 2.47	0.275
Antidepressants, mean ± SD	7.98 ± 3.01	7.75 ± 2.44	0.282
Anxiolytics mean ± SD	8.03 ± 2.38	7.75 ± 2.58	0.250
Anti-inflammatory agents/Analgesics, mean ± SD	7.83 ± 2.45	7.77 ± 2.59	0.430
Anti-inflammatory agents, mean ± SD	7.83 ± 2.45	7.77 ± 2.59	0.430
Analgesics, mean ± SD	7.99 ± 2.44	7.77 ± 2.62	0.312
ba-PWV (m/s)			
Cardiovascular drugs, mean ± SD	13.76 ± 2.37	12.40 ± 2.27	<0.001
Antihypertensives, mean ± SD	13.63 ± 2.44	12.63 ± 2.34	0.004
Antihyperlipidaemics, mean ± SD	13.95 ± 2.06	12.59 ± 2.38	<0.001
Hypoglycaemics, mean ± SD	14.02 ± 1.50	12.74 ± 2.40	0.034
Platelet aggregation inhibitors/Anticoagulants, mean ± SD	14.23 ± 2.76	12.73 ± 2.35	0.020
Antidepressants/Anxiolytics mean ± SD	12.42 ± 2.12	12.89 ± 2.40	0.093
Antidepressants, mean ± SD	12.55 ± 2.49	12.83 ± 2.37	0.231
Anxiolytics mean ± SD	12.48 ± 2.25	12.84 ± 2.40	0.181
Anti-inflammatory agents/Analgesics, mean ± SD	12.51 ± 1.93	12.88 ± 2.51	0.089
Anti-inflammatory agents, mean ± SD	12.51 ± 1.93	12.88 ± 2.51	0.089
Analgesics, mean ± SD	12.91 ± 1.81	12.79 ± 2.43	0.386
CAVI			
Cardiovascular drugs, mean ± SD	7.89 ± 1.19	7.34 ± 1.26	<0.001
Antihypertensives, mean ± SD	7.74 ± 1.17	7.46 ± 1.28	0.077
Antihyperlipidaemics, mean ± SD	7.97 ± 1.18	7.42 ± 1.26	0.004
Hypoglycaemics, mean ± SD	8.13 ± 0.83	7.48 ± 1.27	0.041
Platelet aggregation inhibitors/Anticoagulants, mean ± SD	8.24 ± 0.76	7.47 ± 1.27	0.004
Antidepressants/Anxiolytics mean ± SD	7.23 ± 1.09	7.57 ± 1.30	0.036
Antidepressant drugs, mean ± SD	7.36 ± 1.14	7.53 ± 1.29	0.188
Anxiolytics drugs mean ± SD	7.29 ± 1.21	7.54 ± 1.27	0.116
Anti-inflammatory agents/Analgesics, mean ± SD	7.32 ± 1.17	7.57 ± 1.29	0.070
Anti-inflammatory drugs, mean ± SD	7.32 ± 1.17	7.57 ± 1.29	0.070
Analgesic drugs, mean ± SD	7.61 ± 1.22	7.51 ± 1.28	0.322

Values are means and standard deviations for continuous data, and number and proportions for categorical data. c-IMT, intima–media thickness of common carotid; cf-PWV, carotid-femoral pulse wave velocity; ba-PWV, brachial-ankle pulse wave velocity; CAVI, cardio-ankle vascular index.

*p* value: differences between increase and no increase.

For antidepressants/anxiolytics, only CAVI was higher in participants without increased use (with increase: 7.23 ± 1.09 vs. 7.57 ± 1.30, *p* = 0.036). The anti-inflammatory/analgesic group showed no significant differences overall (*p* > 0.05).

In summary, the greatest vascular alterations were observed in participants with increased cardiovascular drug use.

### 3.4 Relationship between increased drug use and vascular parameters

**Figure 2:**
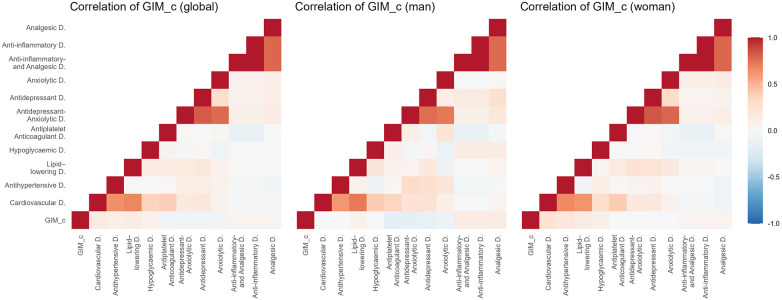
Heatmap of correlation between c-IMT and increased drug use, overall and by sex. GIM_c: c-IMT: intima–media thickness of common carotid.

**Figure 3:**
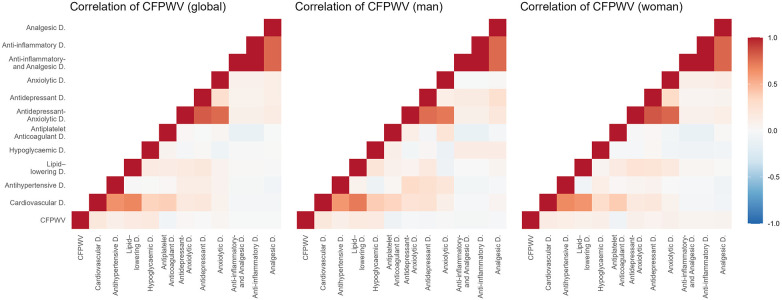
Heatmap of correlation between cf-PWV and increased drug use, overall and by sex. Cf-PWV: carotid-femoral pulse wave velocity.

**Figure 4:**
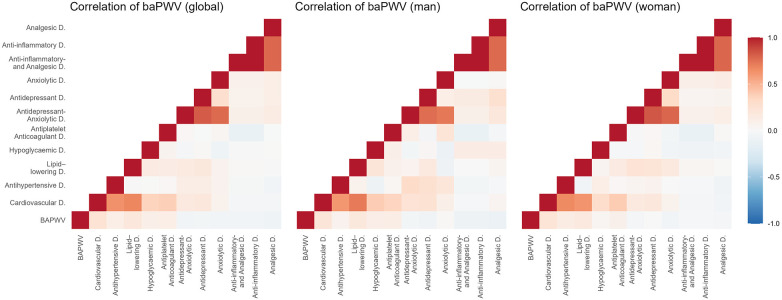
Heatmap of correlation between ba-PWV and increased drug use, overall and by sex. Ba-PWV, brachial-ankle pulse wave velocity.

**Figure 5:**
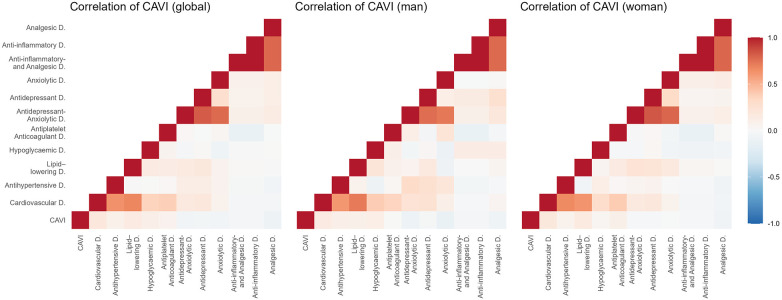
Heatmap of correlation between CAVI and increased drug use, overall and by sex. CAVI, cardio-ankle vascular index.

Figures 2–5 and Supplementary Table S1 show the overall and sex-specific correlations between vascular structure and function measures and increased drug use. Greater use of cardiovascular, antihypertensive and antihyperlipidaemics was positively correlated with c-IMT (r = 0.222, r = 0.151 and r = 0.171, respectively). Taking more cardiovascular, antihypertensive, antihyperlipidaemic and hypoglycaemic drugs was positively correlated with cf-PWV (r = 0.196, r = 0.117, r = 0.153 and r = 0.171, respectively) and ba-PWV (r = 0.256, r = 0.113, r = 0.231, r = 0.146, repectively), and using more cardiovascular and antihyperlipidaemics had a positive correlation with CAVI (r = 0.200). All *p* values ​​were <0.005. No significant correlations were found between increased use of antidepressants/anxiolytics or anti-inflammatories/analgesics and vascular parameters.

**Figure 6:**
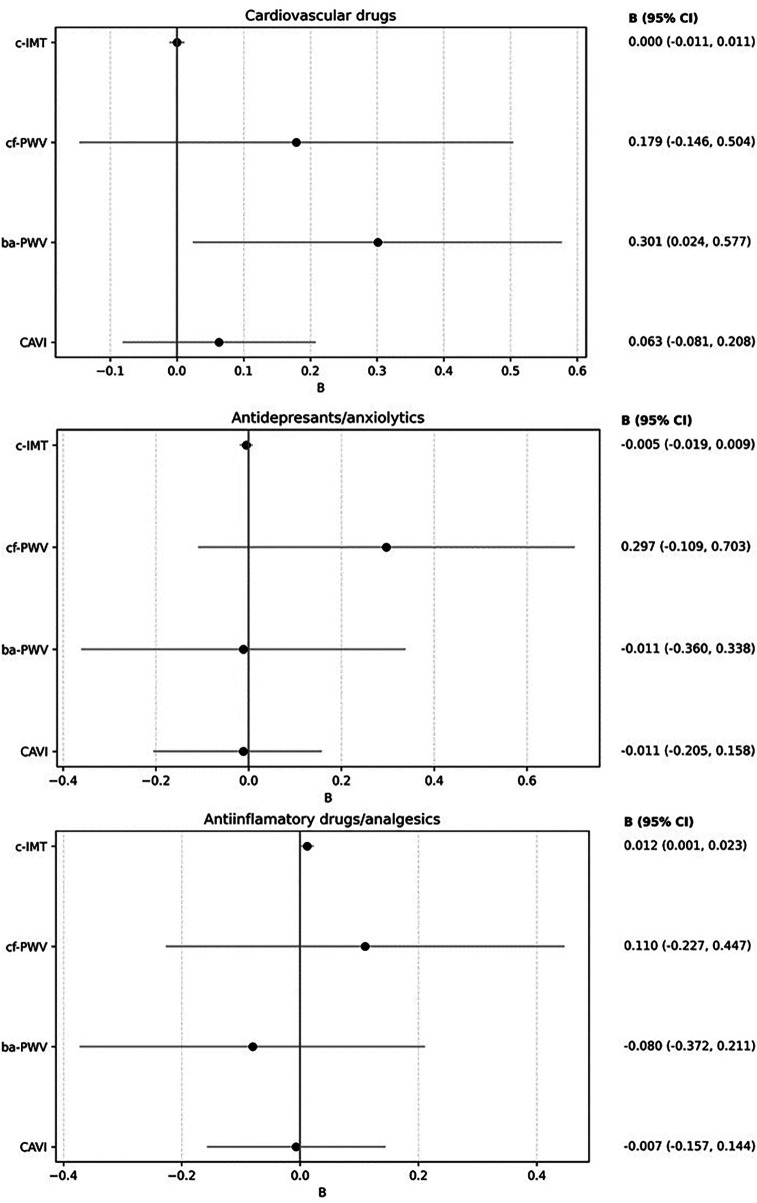
Forest plot: association between increased drug use and vascular parameters. Multiple regression analysis. Dependent variable: “c-IMT”, “cf-PWV”, “ba-PWV” and “CAVI”. Independent variables: increased drug use (cardiovascular drugs, antidepressants/anxiolytics and Anti-inflammatory agents/analgesics). Adjustment variables: age and sex. c-IMT, Intima–media thickness of common carotid; cf-PWV, carotid-femoral pulse wave velocity; ba-PWV, Brachial-ankle pulse wave velocity; CAVI: Cardio-ankle vascular index.

The results of the multiple regression analysis, adjusted for age and sex, are shown in Figure 6 and Supplementary Table S2. Increased use of cardiovascular medications was positively associated with ba-PWV (*β* = 0.301, 95% CI: 0.024–0.577). Increased use of anti-inflammatory/analgesic medications was positively associated with c-IMT (*β* = 0.012, 95% CI: 0.001–0.023). No other associations reached statistical significance.

**Figure 7:**
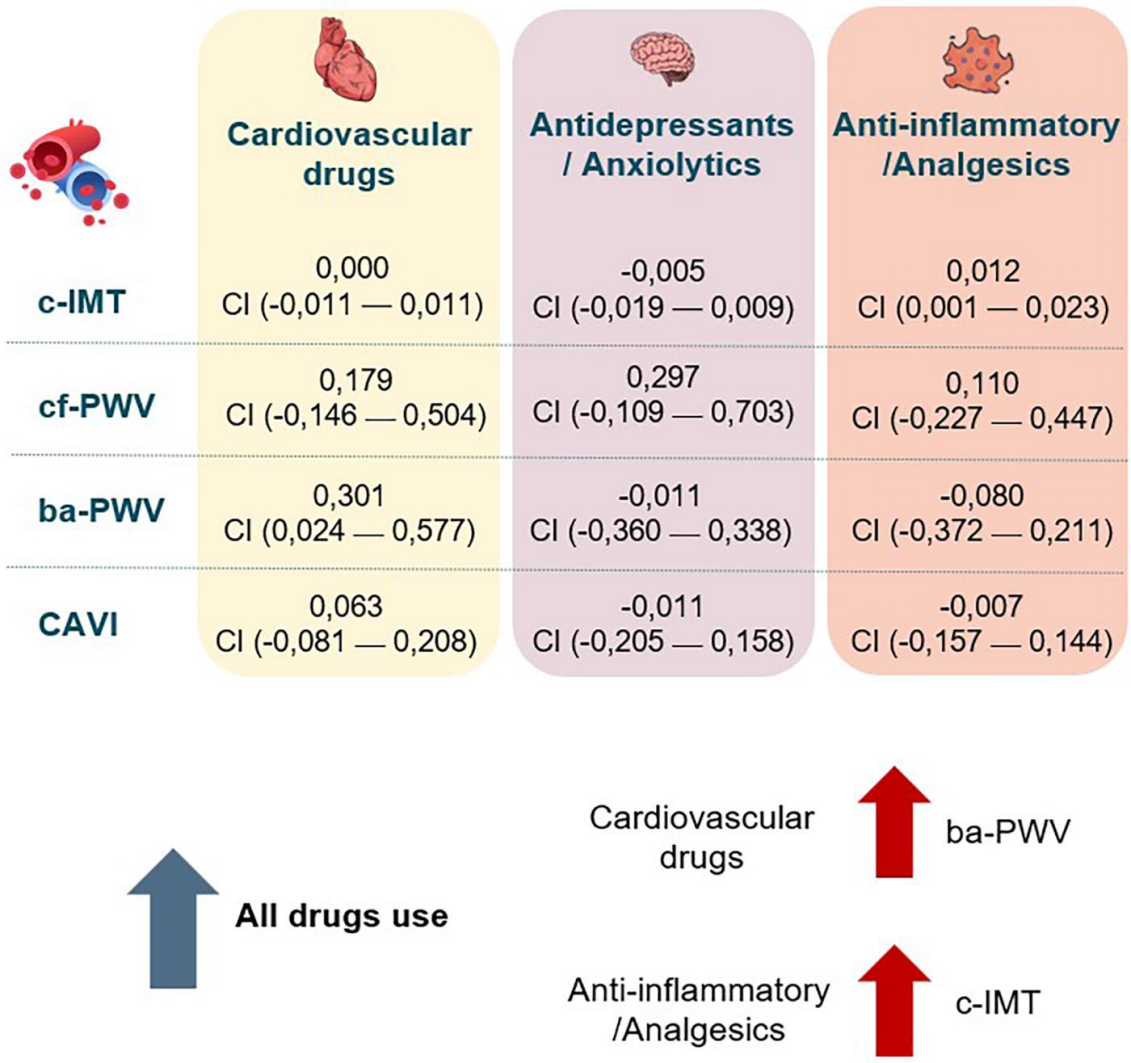
Summary of associations between increased drug use and vascular parameters. c-IMT, Intima–media thickness of common carotid; cf-PWV, carotid-femoral pulse wave velocity; ba-PWV, Brachial-ankle pulse wave velocity; CAVI, cardio-ankle vascular index.

A summary figure illustrating the associations between the increase in drug use and arterial stiffness parameters during LC is provided in Figure 7.

## 4 Discussion

Although several studies have described increased use of medication among patients with LC, there are no detailed analyses of this use and its relationship to vascular health. This study addresses a previously unexplored aspect of increased drug use following the onset of LC and its relationship with vascular structure parameters (determined by c-IMT) and arterial stiffness (by cf-PWV, ba-PWV, and CAVI). While the main findings suggest an increase in the use of all drug classes, positive associations were only found between the increase in cardiovascular drugs and ba-PWV, and between anti-inflammatory/analgesic drugs and c-IMT, after adjusting for age and sex.

### 4.1 Relationship between cardiovascular drug use and vascular parameters

As Figure 1 shows, the bidirectional relationship between LC and cardiovascular risk factors has been reported in numerous studies (55). For example, the study by Tudoran et al. (56) reinforced this association, highlighting that persistent inflammation and comorbidities contribute to diastolic dysfunction in LC. It is thus hardly surprising to observe greater use of cardiovascular medications in this population. Arterial stiffness is an established cardiovascular risk factor, and several studies have identified an association between SARS-CoV-2 infection and elevated cf-PWV (57).

#### 4.1.1 Antihypertensives

In line with previous studies, we found that use of antihypertensive drugs increased after LC symptoms started (12). This can be directly attributed to the appearance of new-onset hypertension after SARS-CoV-2 infection, dysautonomia (58) and the widespread use (especially of beta-blockers) in the treatment of postural orthostatic tachycardia syndrome (POTS) (59). A 65% increase in the risk of new-onset hypertension has been reported after infection compared to non-infected individuals (60). While hypertension produces structural and functional changes in the blood vessels, promoting an increase in arterial stiffness (24), antihypertensive treatment may reverse this effect (36). Several studies have described lower arterial stiffness (independent of the decrease in blood pressure) produced by taking antihypertensives (61), although no such association was found in the present study. This discrepancy could be due to the influence of the different medications: drugs that interact with the renin-angiotensin-aldosterone system and calcium channel blockers have been associated with lower PWV, mediated by vascular smooth muscle relaxation and a higher elastin-to-collagen ratio. Thiazide diuretics have shown a negative relationship with CAVI, but a positive relationship with PWV, mediated by decreased plasma volume and the resulting peripheral vasoconstriction. Beta-blockers on the other hand, widely used by LC patients (as a treatment for POTS and autonomic dysfunction), lack vasodilatory properties and have no effect on vascular health (28, 35). This study did not analyse the differences between each type of antihypertensive drug, which may partly explain these findings.

#### 4.1.2 Antihyperlipidaemics

Patients with LC start using antihyperlipidaemics in connection with the development of dyslipidemia and cardiovascular complications during the course of the disease (62). In line with our results, research in Romania on patients with LC found a doubling in the number of participants with dyslipidemia in the post-pandemic period compared to the pre-pandemic period (63), as well as an increase in the prescription of antihyperlipidaemics (64). Apart from the alterations produced in the lipid profile, the need for secondary treatment of cardiovascular complications present in LC, such as cardiovascular events, has also increased the use of antihyperlipidaemics (13).

Both dyslipidemia and LC can alter vascular structure. A recent study has shown an increase in c-IMT in patients with LC (65). As with antihypertensive drugs, the level of vascular protection of antihyperlipidaemics varies across groups and doses. High-dose statins and, especially, the use of ezetimibe or proprotein convertase subtilisin/kexin type 9 inhibitors (iPCSK9) were associated with lower PWV and c-IMT, while fibrates showed no correlation (66, 67). The differences may reflect the lack of subgroup analysis. In addition, we analysed the start of antihyperlipidaemics use from the onset of LC, so the prevalence of high-dose statin, ezetimibe or iPCSK9 use, is lower than in cases of long-established hypertension.

#### 4.1.3 Hypoglycaemic agents

Consistent with this study, an increase in DM has been observed in patients with LC (7). This increase in turn has led to many patients starting hypoglycaemic therapy. The presence of DM-induced microvascular disease is related to the severity of the infection (68) and to the alteration of the vascular structure and arterial stiffness (69).

All groups of hypoglycaemics were linked to a reduction in PWV through the activation of eNOS (28). The influence on arterial stiffness is greater in the new antidiabetics [sodium-glucose cotransporter-2 inhibitors (iSGLT-2), dipeptidyl peptidase-4 inhibitors (iDPP-4) or glucagon-like peptide-1 (GLP-1) receptor agonists], which could explain their effects on cardiovascular level (70). The positive correlation between increased hypoglycaemics use and vascular parameters in the present study demonstrates the multifactorial nature of vascular damage in patients with LC. In line with our findings, we have not found any research linking use of antihyperlipidaemics or DM with vascular structure (c-IMT).

#### 4.1.4 Antiplatelet and anticoagulant agents

Starting antiplatelet and anticoagulant drug use in patients with LC is a response to the persistent prothrombotic state (71). Nevertheless, antithrombotic treatment in LC is not yet well established (72). A reduction in peripheral arterial stiffness has been demonstrated with the intake of acetylsalicylic acid (73), purinergic receptor P2Y12 inhibitors (74) and anticoagulants (75).

In short, in contrast to previous studies reporting a reduction in arterial stiffness with the use of cardiovascular drugs, this study shows a positive association between the use of these drugs and arterial stiffness. In addition to the individual characteristics of each medication group, these discrepancies can be explained by the relationship between active and passive arterial stiffness. Active arterial stiffness is mediated by vascular smooth muscle making it dynamic and reversible, while passive arterial stiffness is mediated by collagen and vascular calcification and is therefore permanent (76). Cardiovascular drugs can reverse active arterial stiffness, but not passive stiffness (77). In the case of LC chronic inflammation produces irreversible changes in the vascular wall, promoting collagen deposition. This means that arterial stiffness is not reversible with drug use, which explains why this study shows higher levels of arterial stiffness with increased drug use: the damage caused by LC exceeds the capacity for pharmacological reversibility. This highlights the multifactorial and potentially irreversible nature of vascular damage in LC. In addition, LC-related vascular injury also involves microvascular dysfunction (particularly endothelial glycocalyx degradation and microclot formation) which recent studies have identified as key pathophysiological mechanisms (78). Although the present analysis focuses on macrovascular parameters, these microvascular alterations are complementary and help contextualize the vascular impairment observed in our cohort.

### 4.2 Relationship between the use of antidepressants and anxiolytics and vascular parameters

The psychological impact of the pandemic and the presence of persistent symptoms have contributed to worsening mental health in patients with LC. In line with this research, numerous studies have found an increase in the use of antidepressants and anxiolytics after the pandemic (16, 79). However, there is a lack of studies in patients with LC.

There are studies that have demonstrated an increase in c-IMT and arterial stiffness in depressive or anxiety disorders (80, 81). Consistent with our data (negative association, albeit not significant), several studies have demonstrated the beneficial effect of SSRI on arterial stiffness and carotid atherosclerosis (82), mediated by a reduction in proinflammatory cytokines (37). However, dual serotonin and norepinephrine reuptake inhibitor increase arterial stiffness through adrenergic activation (83). We have not found any studies that assess this relationship with anxiolytic drugs.

### 4.3 Relationship between the use of anti-inflammatory and analgesic drugs and vascular parameters

The increasing use of analgesics in LC development reflects the lack of specificity of the symptoms. Several studies highlight an increase in the use of analgesic drugs to mitigate persistent pain (84).

Although the anti-inflammatory drugs are generally associated with reduced arterial stiffness (28), this effect was not observed for analgesics. The present study found no differences in arterial stiffness, which could be due to the unification of both pharmacological groups into one. Furthermore, in contrast to the data from this study, a decrease in c-IMT was found with anti-inflammatory treatment in patients with rheumatoid arthritis (85). Nevertheless, one study supports the data we obtained, showing worse vascular health in patients with opioid use (86).

### 4.4 Limitations and strengths

This study has several limitations: 1. As a cross-sectional study, causation cannot be inferred; 2. The analysis by sex may be misleading, as the sexes are not represented equally; 3. Change in drug use was analysed by drug group, not individually. The study also has strengths: 1. It offers a unique and detailed perspective on the increase in drug use in patients with LC; 2. The updated WHO definition of LC was applied; 3. The analysis of vascular health was performed by trained and validated researchers following a standardised protocol; 4. The results are adjusted for age and sex, which avoids confounding factors; 5. We consider that the results have important clinical implications and lay the foundation for further research.

## 5 Conclusion

Drug use increased from 2019 to the time of inclusion in the study. While the increase in cardiovascular drugs had a positive association with ba-PWV, increased anti-inflammatory/analgesic drug use was positively linked with c-IMT in subjects diagnosed with LC.

## Data Availability

The datasets presented in this study can be found in online repositories. The names of the repository/repositories and accession number(s) can be found on ZENODO under the DOI: 10.5281/zenodo.14282873 (https://zenodo.org/records/14282873).
