# Supplementary material for: Association between cardiovascular, psychotropic and anti-inflammatory/analgesic drug use and vascular dysfunction in individuals with long COVID. BioICOPER study
Source: Front Cardiovasc Med. 2026 Jan 12;12:1691153. doi: 10.3389/fcvm.2025.1691153 (PMC12832891; doi:10.3389/fcvm.2025.1691153)
Supplement: Supplementary file 1 [file Table1.docx]

**Supplementary Material**

**Table 1S.** Correlation between increased medication use and vascular parameters, overall and by sex.

|  | **c-IMT** | | |
| --- | --- | --- | --- |
|  | **Global** | **Women** | **Men** |
| **Cardiovascular drugs** | 0.222* | 0.249* | 0.105 |
| Antihypertensives | 0.151* | 0.201* | 0.028 |
| Antihyperlipidaemics | 0.171* | 0.133 | 0.164 |
| Hypoglycaemics | 0.097 | 0.092 | 0.034 |
| Platelet aggregation inhibitors /Anticoagulants | 0.019 | 0.049 | -0.056 |
| **Antidepressants/Anxiolytics** | -0.062 | 0.005 | -0.175 |
| **Antidepressants** | -0.081 | -0.023 | -0.168 |
| **Anxiolytics** | -0.028 | -0.007 | -0.036 |
| **Anti-inflammatory agents/Analgesics** | 0.039 | 0.054 | 0.108 |
| **Anti-inflammatory agents** | 0.011 | 0.046 | 0.074 |
| **Analgesics** | 0.040 | 0.025 | 0.096 |
|  | **cf-PWV** | | |
|  | **Global** | **Women** | **Men** |
| **Cardiovascular drugs** | 0.196* | 0.138* | 0.242* |
| Antihypertensives | 0.117* | 0.108 | 0.128 |
| Antihyperlipidaemics | 0.153* | 0.085 | 0.213* |
| Hypoglycaemics | 0.171* | 0.128 | 0.197 |
| Platelet aggregation inhibitors /Anticoagulants | -0.057 | -0.061 | -0.092 |
| **Antidepressants/Anxiolytics** | 0.031 | 0.084 | 0.003 |
| **Antidepressants** | 0 | 0.040 | -0.009 |
| **Anxiolytics** | 0.046 | 0.068 | 0.054 |
| **Anti-inflammatory agents/Analgesics** | 0.036 | 0.077 | 0.029 |
| **Anti-inflammatory agents** | 0.019 | 0.073 | 0.027 |
| **Analgesics** | 0.009 | 0.025 | -0.015 |
|  | **ba-PWV** | | |
|  | **Global** | **Women** | **Men** |
| **Cardiovascular drugs** | 0.265* | 0.228* | 0.270* |
| Antihypertensives | 0.113* | 0.115 | 0.084 |
| Antihyperlipidaemics | 0.231* | 0.189* | 0.236* |
| Hypoglycaemics | 0.146* | 0.079 | 0.157 |
| Platelet aggregation inhibitors /Anticoagulants | 0.098 | 0.072 | 0.144 |
| **Antidepressants/Anxiolytics** | -0.089 | -0.058 | -0.081 |
| **Antidepressants** | -0.070 | -0.039 | -0.091 |
| **Anxiolytics** | -0.062 | -0.072 | 0.033 |
| **Anti-inflammatory agents/Analgesics** | -0.013 | 0.043 | -0.024 |
| **Anti-inflammatory agents** | -0.050 | 0.032 | -0.090 |
| **Analgesics** | 0.035 | 0.058 | 0.034 |
|  | **CAVI** | | |
|  | **Global** | **Women** | **Men** |
| **Cardiovascular drugs** | 0.200* | 0.179* | 0.192 |
| Antihypertensives | 0.076 | 0.064 | 0.102 |
| Antihyperlipidaemics | 0.161* | 0.176* | 0.097 |
| Hypoglycaemics | 0.112 | 0.035 | 0.116 |
| Platelet aggregation inhibitors /Anticoagulants | 0.080 | 0.106 | 0.010 |
| **Antidepressants/Anxiolytics** | -0.086 | -0.041 | -0.148 |
| **Antidepressants** | -0.058 | -0.043 | -0.041 |
| **Anxiolytics** | -0.050 | -0.012 | -0.108 |
| **Anti-inflammatory agents/Analgesics** | -0.040 | -0.039 | 0.008 |
| **Anti-inflammatory agents** | -0.090 | -0.068 | -0.076 |
| **Analgesics** | 0.050 | 0.052 | 0.066 |

c-IMT: Intima–media thickness of common carotid; cf-PWV: carotid-femoral pulse wave velocity; ba-PWV: Brachial-ankle pulse wave velocity; CAVI: Cardio-ankle vascular index.

* p < 0.05

**Table 2S.** Association between vascular parameters and cardiovascular drugs, antidepressants/anxiolytics, and anti-inflammatory agents/analgesics. Multiple regression analysis.

|  | **B** | **(95%) CI** | | **p value** |
| --- | --- | --- | --- | --- |
| **Cardiovascular drugs** | | | | |
| c-IMT (mm) | 0 | -0.011 | 0.011 | 0.961 |
| cf-PWV (m/s) | 0.179 | -0.146 | 0.504 | 0.279 |
| ba-PWV (m/s) | 0.301 | 0.024 | 0.577 | 0.033 |
| CAVI | 0.063 | -0.081 | 0.208 | 0.389 |
| **Antidepressants/anxiolytics** | | | | |
| c-IMT (mm) | -0.005 | -0.019 | 0.009 | 0.504 |
| cf-PWV (m/s) | 0.297 | -0.109 | 0.703 | 0.151 |
| ba-PWV (m/s) | -0.011 | -0.360 | 0.338 | 0.952 |
| CAVI | -0.011 | -0.205 | 0.158 | 0.797 |
| **Anti-inflammatory agents/analgesics** | | | | |
| c-IMT (mm) | 0.012 | 0.001 | 0.023 | 0.040 |
| cf-PWV (m/s) | 0.110 | -0.227 | 0.447 | 0.521 |
| ba-PWV (m/s) | -0.080 | -0.372 | 0.211 | 0.588 |
| CAVI | -0.007 | -0.157 | 0.144 | 0.932 |

Multiple regression analysis using ‘vascular parameters’ as dependent variable and ‘increase of drugs’ consume’, ‘age’ and ‘sex’ as independent variables. c-IMT: Intima–media thickness of common carotid; cf-PWV: carotid-femoral pulse wave velocity; ba-PWV: Brachial-ankle pulse wave velocity; CAVI: Cardio-ankle vascular index.
