# Supplementary figures and images for: Association between cardiovascular, psychotropic and anti-inflammatory/analgesic drug use and vascular dysfunction in individuals with long COVID. BioICOPER study
Source: Front Cardiovasc Med. 2026 Jan 12;12:1691153. doi: 10.3389/fcvm.2025.1691153 (PMC12832891; doi:10.3389/fcvm.2025.1691153)

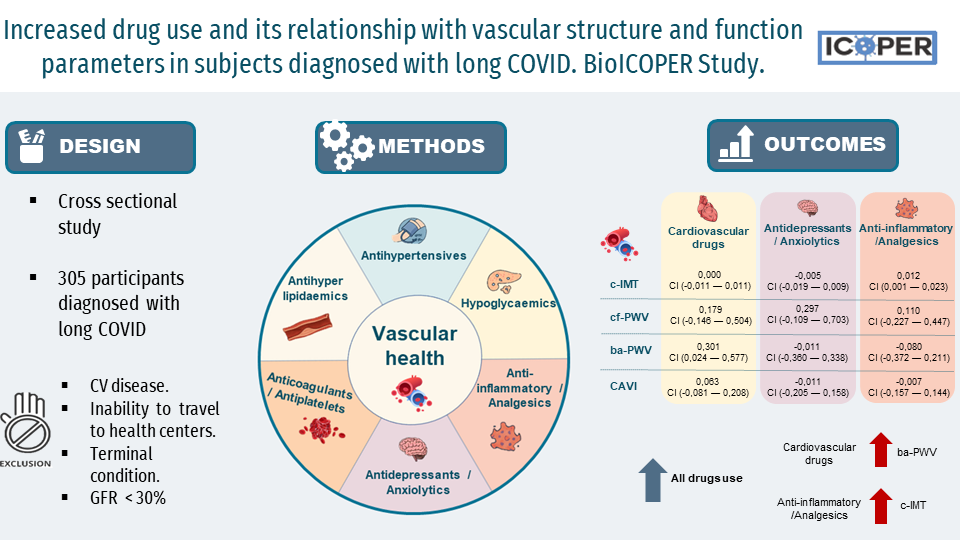

Supplement: Supplementary file 2 [file Image1.png]
